# Standardization and Evaluation of the LAMP Technique for the Diagnosis of Canine Visceral Leishmaniasis in Conjunctival Swab Samples Using DNA Extracted by a Silica Column and Boiling

**DOI:** 10.3390/tropicalmed9110277

**Published:** 2024-11-14

**Authors:** Isabela C. S. Santos, Daniel M. Avelar, Luciana F. C. Miranda, Cintia X. de Mello, Lucas Keidel, Maria Inês F. Pimentel, Luanna S. Ventura, Aline Fagundes, Fernanda N. Santos, Liliane F. A. Oliveira, Shanna A. Santos, Sandro Antonio Pereira, Rodrigo C. Menezes, Andreza P. Marcelino

**Affiliations:** 1 Leishmaniasis Clinical Research and Surveillance Laboratory, Evandro Chagas National Institute of Infectious Diseases (INI), Oswaldo Cruz Foundation Fiocruz, Rio de Janeiro 21040-900, Rio de Janeiro, Brazil; isabela.cordeiro@ini.fiocruz.br (I.C.S.S.); luciana.freitas@ini.fiocruz.br (L.F.C.M.); maria.pimentel@ini.fiocruz.br (M.I.F.P.); aline.fagundes@ini.fiocruz.br (A.F.); fernanda.nunes@ini.fiocruz.br (F.N.S.); liliane.fatima@ini.fiocruz.br (L.F.A.O.); shanna.santos@ini.fiocruz.br (S.A.S.); 2 Clinical Research and Public Policies in Infectious and Parasitic Diseases Laboratory, René Rachou Institute, Oswaldo Cruz Foundation (Fiocruz), Belo Horizonte 30190-002, Minas Gerais, Brazil; daniel.avelar@fiocruz.br; 3 Interdisciplinary Medical Research Laboratory, Instituto Oswaldo Cruz (IOC), Oswaldo Cruz Foundation Fiocruz, Rio de Janeiro 21040-900, Rio de Janeiro, Brazil; cintia.mello@ioc.fiocruz.br; 4 Laboratory of Clinical Research on Dermatozoonosis in Domestic Animals, Evandro Chagas National Institute of Infectious Diseases (INI), Oswaldo Cruz Foundation (Fiocruz), Rio de Janeiro 21040-900, Rio de Janeiro, Brazil; lucas.keidel@ini.fiocruz.br (L.K.); sandro.pereira@ini.fiocruz.br (S.A.P.); rodrigo.menezes@ini.fiocruz.br (R.C.M.)

**Keywords:** canine visceral leishmaniasis, molecular biology, diagnosis, conjunctival swab

## Abstract

The diagnosis of canine visceral leishmaniasis (CVL) presents a challenge due to a variety of non-specific clinical signs. The available tests have low sensitivity. This study aimed to standardize and evaluate the loop-mediated isothermal amplification technique with K26 target (K26-LAMP) for diagnosis of CVL in conjunctival swab (CS) DNA samples extracted through a silica column commercial kit (SW-kit) and boiling (SW-DB) and to compare sensitivity with conventional PCR (kDNA-cPCR) and quantitative real-time PCR (18S-qPCR). Clinical samples of CSs were collected from 54 dogs after reactive serology tests. Positive parasitological and/or histological tests were used as inclusion criteria for a sensitivity analysis. A total of 79.2% (43/54) of dogs without clinical signs or with mild, moderate, or severe clinical signs were included in the study. The sensitivity results of K26-LAMP, kDNA-cPCR, and 18S-qPCR were 72.1%, 81.4%, and 80.5% with the SW-kit and 97.2%, 95.2%, and 57.1% with SW-DB, respectively. In all techniques, the proportion of positives was higher in the group with severe clinical disease, with statistically significant differences in the K26-LAMP and 18S-qPCR techniques being seen with the SW-kit. The results obtained with LAMP for CS samples are promising and its performance is similar to other techniques.

## 1. Introduction

Leishmaniasis are neglected diseases caused by protozoa of the genus *Leishmania* with different clinical presentations, including visceral leishmaniasis (VL) and cutaneous leishmaniasis (CL). The main etiological agent of VL in Brazil is *Leishmania (Leishmania) infantum.* Transmission occurs during the bite female sandflies (mainly *Lutzomyia longipalpis* in Brazil) on their hosts, with domestic dogs being the main urban reservoir of the disease [1].

Around 80 countries are endemic for VL, and Brazil is responsible for over 90% of the reported cases in Latin America [2]. In the state of Rio de Janeiro, human VL was first reported in the 1970s, and canine VL (CVL) has been reported ever since [3].

In dogs, the infection is usually chronic and multisystemic, and the clinical signs of the disease include weight loss, skin lesions (such as alopecia and dermatitis), onychogryphosis, ophthalmic lesions (conjunctivitis and/or blepharitis), and lymphadenomegaly, among others [4]. However, many animals remain no clinical signs for a long time or only show non-specific clinical signs, such as skin disorders [5].

The most used tests to diagnose CVL in Brazil are serological tests, rapid dual-platform immunochromatographic tests (RT DPP) and enzyme-linked immunosorbent assay (ELISAs). Their performance is questionable, especially in dogs without clinical signs [6,7]. Parasitological tests such as direct examination and the culture of material obtained through aspiration from bone marrow or lymph nodes are more invasive techniques, are centralized in reference laboratories, and have low sensitivity despite their high specificity [8].

For the diagnosis of CVL, molecular techniques such as polymerase chain reactions (PCRs) have satisfactory performance and allow for the use of different types of samples [9,10], including less invasive samples such as conjunctival swabs [11]. However, these tests are commonly restricted to research centers [12] due to the lack of diagnostic kits, standardization of the technique, and because they require a complex infrastructure to be carried out. The loop-mediated isothermal amplification (LAMP) molecular technique may be a simpler alternative, capable of amplifying large quantities of DNA under isothermal conditions within 30–60 min. Furthermore, its results can be read with the naked eye through turbidity or the addition of dyes/fluorescence, such as SybrGreen, malachite green, or pH change [13,14].

The extraction of DNA from swab samples through the boiling method (DB) associated with LAMP, conventional PCR (cPCR), and real-time PCR (qPCR) has already been evaluated for the diagnosis of CL in humans, proving to be useful in areas with limited infrastructures [15,16]. However, this method of extraction of DNA has not yet been evaluated for the diagnosis of CVL. Therefore, this study aims to standardize and evaluate the K26-LAMP technique for the diagnosis of CVL in conjunctival swab DNA samples extracted through silica column or boiling (DB) and to compare its sensitivity with the *k* DNA-cPCR and 18S-qPCR techniques for the diagnosis of CVL.

## 2. Materials and Methods

### 2.1. Study Population and Inclusion Criteria 

Barra Mansa is a city located in the southern region of the state of Rio de Janeiro (RJ), Brazil, 110 km from the capital of RJ. The city is endemic for human and canine VL caused by *L. (L.) infantum* and transmitted by *Lu. longipalpis*. Fifty-four dogs from this city with reactive serology (VL Rapid Immunochromatographic Test-RT DPP/Bio-Manguinhos^®^ and VL Immunoenzymatic Assay/Bio-Manguinhos^®^) for leishmaniasis with or without clinical signs were investigated from 2020 to 2023. Parasitological (cytological examination and culture) and/or histological (histopathology and immunohistochemistry) tests on bone marrow, lymph node aspirate, and skin or spleen samples were used as reference standards for analyzing the sensitivity of the molecular techniques. The inclusion criteria for the study were dogs with positive results in at least one parasitological test.

### 2.2. Clinical Examination

The dogs were evaluated by veterinarians from the Laboratory of Clinical Research in Dermatozoonosis in Domestic Animals (LapClinDermzoo/INI), and the information on the health of the animals was checked during anamnesis and clinical examination (inspection of skin, mucous membranes, external genitalia, palpation of lymph nodes and abdominal organs) and included in appropriate clinical records.

Clinical signs were classified according to a score [4] as follows: absent/not applicable (0); present (1); intense (2). The presence or intensity of the clinical signs might have been recorded as “local”, “generalized”, and/or other respective terms referring to the intensity of the clinical sign. The dogs were classified according to the sum of their clinical scores as follows (Table 1):

### 2.3. Sample Collection

After clinical examination, the dogs were sedated by intramuscular administration of ketamine hydrochloride 10% (5 mg/kg), acepromazine maleate 1% (0.02 mg/kg), and midazolam hydrochloride 5 mg/mL (0.3 mg/kg). During sedation, a bone marrow (BM) aspiration was performed in the sternum region. The obtained sample was smeared on a microscope slide for cytological examination and an aliquot was also collected in a tube with EDTA to create a culture of the parasite. After collecting this sample, the animal was euthanized with an overdose of sodium thiopental and potassium chloride intravenously. Immediately after euthanasia, a necropsy was carried out for macroscopic examination of the organs and collection of biological samples to detect *Leishmania* and the identification of the species. Fragments of intact skin were aseptically collected using a 3 mm sterile punch from the scapular region and fragments of the spleen and popliteal lymph node were aspirated by a fine needle (AL). They were aliquoted into sterile plastic microtubes containing 1 mL of sterile saline solution with penicillin 1200 IU, streptomycin 1000 µg, and 5′fluorocytocin 100 µg and stored at 4 °C for a parasitological culture. Additionally, spleen imprints were made on a microscope slide for cytological examination. Additional fragments of intact skin and spleen were collected and aliquoted into sterile dry plastic microtubes, then stored in microplastics with 10% buffered formalin for histopathological and immunohistochemical analysis. In both eyes, exfoliative conjunctival epithelial cells were obtained using two sterile swabs per animal for a subsequent molecular analysis. The swabs were placed in sterile microtubes containing sterile 0.9% saline solution and kept at −20 °C until molecular tests were carried out.

### 2.4. Parasitological Tests

The following tests were carried out: 1. cytological examination under an optical microscope of bone marrow smears and/or spleen imprints fixed with methanol and stained with Giemsa; 2. cultures of spleen, skin, lymph node, and bone marrow aspirates in Novy-–MacNeal–Nicolle (NNN) + Schneider biphasic media, plus antibiotic and antifungal media, according to protocols standardized by the Leishmaniasis Clinical Research and Surveillance Laboratory [17,18]. Cultures were monitored weekly for the growth of promastigotes of *Leishmania* spp. for up to 30 days (4 weeks/4 readings). Positive samples were inoculated in culture bottles containing the same biphasic media mentioned above to obtain a sufficient parasite mass for cryopreservation [18]. In dogs with negative results in terms of culture and cytological examination, histopathology and immunohistochemistry techniques were carried out to detect amastigotes of *Leishmania* spp. For both techniques, fragments of skin, spleen, jejunum, and cecum were fixed in 10% formalin and embedded in paraffin. The histological sections were stained with hematoxylin–eosin. For immunohistochemistry, histological tissue sections were processed and incubated with rabbit polyclonal anti—*Leishmania* serum (1:500) according to Boechat et al. [19]. For the detection of *Leishmania* amastigotes, the HiDef Detection HRPTM Polymer System (Cell Marque, Rocklin, CA, USA) was used according to the manufacturer’s recommendations.

### 2.5. Molecular Tests

DNA extraction from clinical samples from one of the swabs (SW-kit) was performed using a silica column, the PureLink^®^ Genomic DNA Mini Kit (Invitrogen by Thermo Fisher Scientific- Waltham, MA, USA), according to the manufacturer’s guidelines. In the other swab sample (SW-DB), extraction with the Direct Boil method was used [16], known as boiling. After extraction, DNA was stored at −20 °C until use. The extracted DNA was measured and the purity ratio of 260/280 was established by spectrophotometry on the NanoDrop 2000 equipment (Thermo Fisher Scientific—Waltham, MA, USA).

### 2.6. DNA Amplification

For DNA amplification through cPCR, qPCR and LAMP, protocols adapted from kDNA-cPCR [20], 18S-qPCR [21], GAPDH [22], and K26-LAMP were used [23]. DNA extracted from the reference strain of *L. (L.) infantum* (MHOM/BR/1974/PP75) was used as the positive control (PC) and, for preparing a standard curve [24] and a PCR/LAMP reagent mix without DNA, an extraction control (extraction reagents) and other negative controls from healthy dogs were used as negative controls (NCs).

### 2.7. LAMP Standardization

LAMP reactions were standardized using the WarmStart ^®^ commercial colorimetric mix kit (New England Biolabs, Inc., Ipswich, MA, USA). Serial dilutions of 100 picograms to one fentogram of DNA from the *L. (L.) infantum* reference sample (MHOM/BR/1974/PP75) were used to determine the LAMP detection limits. Incubation times between 30 and 60 min were tested in a water bath (Novatécnica, model NT 248—Piracicaba, São Paulo, Brazil), with observations being made every 10 min during this period. The results, according to visual reading by two independent observers, were noted immediately after the test. Observer 1 was a biologist experienced in molecular biology diagnostics who was trained to perform and interpret LAMP by the René Rachou Institute, Fiocruz. Observer 2 was a biomedicine student, also experienced in molecular biology diagnostics, trained by observer 1. Each observer carried out their analysis individually, and, after the second observer’s reading, the results were compared and discussed between them. For samples with divergent results between readers, a third observer analyzed the result using an archived image of the test. Observer 3 was a veterinarian with experience in validating diagnostic tests who was trained by the René Rachou Institute, Fiocruz. Agarose gels were run on all the LAMP tests to confirm the visual reading results.

### 2.8. Leishmania Species in Clinical Samples

The species were characterized by using the multilocus enzyme electrophoresis technique (MLEE), with the enzyme systems glucose-6-phosphate dehydrogenase (G6PDH), phosphoglucose isomerase (GPI), nucleoside hydrolase (NH), phosphoglucomutase (PGM), and 6-phosphogluconate dehydrogenase (6PGDH), according to a standardized protocol [18]. Parasite masses obtained from positive cultures were used. The banding profiles of the *Leishmania* species isolated from the dogs were compared with the profiles of the reference strains of *L. (L.) infantum* (MHOM/BR/1974/PP75) and *L. (Viannia) braziliensis* (MHOM/BR/1975/M2903). For the samples that did not grow in culture and/or did not show adequate results in the MLEE technique, PCR-RFLP characterization was carried out according to the protocol of Graça et al. [25]. For PCR-RFLP, DNA was extracted from the *Leishmania* culture using the PureLink Genomic DNA Minikit (Thermo Fisher Scientific, Waltham, MA, USA), obtained from bone marrow. The obtained restriction profiles (digestion with the enzymes HAEIII and BstuI) were compared with those obtained with the same reference strains used in the biochemical characterization-MLEE and *L. (V.) guyanensis* strain (MHOM/BR/1975/M4147), and they were analyzed in a 6% polyacrylamide gel stained with silver nitrate.

### 2.9. Statistical Analysis

The analyses were carried out using the statistical software Statistical Package for Social Sciences version 19.0 (IBM Corp, Armonk, NY, USA). The statistical analysis was carried out in two stages: (1) analysis of clinical and laboratory variables; (2) analysis of the performance of the test to be evaluated (clinical sensitivity) compared to the reference standard. Frequencies were calculated for categorical variables and measures of central tendency (median, minimum and maximum) for continuous variables. The sensitivity of the molecular techniques was calculated for both extraction methods. The sensitivities of the extraction methods in the different molecular techniques were compared using the McNemar test. A Mann–Whitney test was used to compare continuous variables that did not have a normal distribution. The association between the clinical score and the sensitivities of the molecular techniques was investigated using a Fisher’s exact test. The confidence interval used was 95% with a significant level of 5% (*p* < 0.05). To compare the DNA yield and quality obtained from the swab samples and the median 18S rDNA (18S-qPCR) cycle threshold (Ct) values of samples within the two evaluated extraction protocols, we used a non-parametric Wilcoxon test.

### 2.10. Ethical Aspects

This study was approved by the Ethics Committee for the Use of Animals of the Oswaldo Cruz Foundation (CEUA/Fiocruz; license number LW-19/20).

## 3. Results

In the cytological examination, the positivity rate was 63% (29/46), with 14% (8/54) of samples being lost due to insufficient material for the technique and/or failure in the collection process. The culture positivity rate was 72% (39/54). Three dogs that were negative in the direct examination and culture tests were positive in immunohistochemistry. The overall positivity rate of the parasitological tests (combination of tests) was 79.2% (43/54), and these 43 dogs were admitted to the study and used to calculate the sensitivity of the molecular techniques. The clinical signs observed in the dogs are described in Table 2.

The most frequently found clinical signs were dermatitis (and/or ulcers), alopecia, poor body condition, ophthalmic lesions, and onychogryphosis. Among the visceral signs, splenomegaly and lymphadenopathy were the most frequent. Regarding the clinical score, 26 (60.4%) dogs were classified in group 2 (mild/moderate clinical signs), 16.0 (37.2%) were classified in group 3 (severe clinical signs), and one dog (23.2%) was classified in group 1 (no clinical signs).

**Figure 1:**
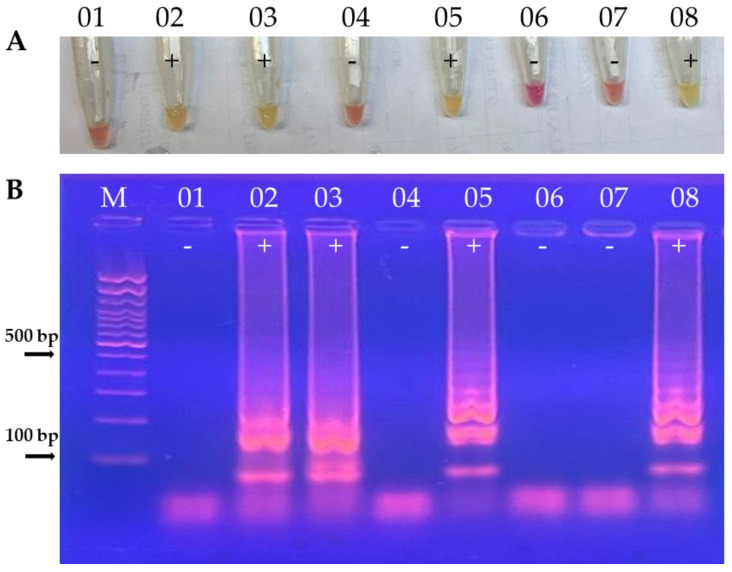
(**A**). Visual detection of K26-LAMP assay results with clinical samples (swab with DNA extracted through boiling method) from dogs with CVL (top row). Tubes 1 to 5: clinical samples; tube 6: healthy dog negative control; tube 7: negative control of the reaction mixture with water; tube 8: positive control of the reference strain of *L. (L.) infantum*. (**B**) K26-LAMP products studied by agarose gel electrophoresis (bottom row) referring to the tubes in the top row. Lane M, 100 bp DNA ladder; lanes 1 to 5: clinical samples; lane 6: healthy dog negative control; lane 7: negative control of the reaction mixture with water; lane 8: positive control of the reference strain of *L. (L.) infantum*, (+): positive result; (-): negative result.

The K26-LAMP reaction was standardized as follows: 1.6 μM of primers FIP and BIP; 0.2 μM of F3 and B3; 0.8 μM of FLP and BLP; 0.8 M of betaine; 40 minutes of incubation for amplification. The reactions were carried out in a final volume of 25 μL, using 12.5 μl of WarmStart^®^ mix and 10–50 ng of DNA. The analytical sensitivity of the test was 100 fg. Figure 1 illustrates some SW-DB K26-LAMP results.

Quantification of DNA (ng/µL) by the nanodrop method, featuring a purity ratio of 260/280 and the cycle threshold (Ct) values of the qPCR reactions per clinical sample, showed statistically significant differences when comparing the DNA samples extracted by SW-DB and the SW-kit, as described in Table 3.

The overall sensitivity results of the molecular techniques are detailed in Table 4.

During the standard curve validation, we obtained an average efficiency curve of 94% [SD: 2.9] and a coefficient correlation (R2) of 0.996 [SD: 0.001] for the 18SrDNA curve and an average efficiency curve of 103,3% [SD: 3.6] and a coefficient correlation (R2) of 0.984 [SD: 0.005] for the GAPDH curve. The threshold value was set at 0.05. The melting temperature was 80.51˚C [SD: 0.0] for 18SrDNA and 80.58˚C [SD: 0.05] for GAPDH. Severe clinical cases had a lower median Ct of 22.35 (17.41–30.60) than mild/moderate clinical cases, whose median Ct was 29.70 (20.44–32.26) with the SW-kit. In SW-DB, the median Ct 27.08 (20.08–33.04) in severe clinical cases was lower than in mild/moderate clinical cases, whose median Ct was 31.49 (27.37–33.52) *p* = 0.001; 0.005. Table 5 shows the proportion of positivity among the groups for each molecular technique.

Twelve dogs had ophthalmological alterations, and all the molecular tests were positive, except for one LAMP sample, the DNA of which was extracted using a commercial kit.

Thirty-seven dogs were characterized as infected with *L. (L.) infantum,* with 19 being identified by the MLEE technique and 18 by PCR-RFLP. It was not possible to identify the species of *Leishmania* from six dogs using the mentioned techniques.

## 4. Discussion

According to our results, the LAMP technique for diagnosing CVL is promising and allows the use of different DNA extraction methods (silica column extraction and boiling extraction), has a good performance in terms of using ocular conjunctival samples collected by cotton swab, and is comparable to cPCR and qPCR techniques.

The most frequent clinical findings of the affected dogs were skin lesions, poor body condition, skin scaling, onychogryphosis, and splenomegaly, and these corroborate the clinical manifestations described in the literature [4,5,26,27]. The fact that there was only one dog with no clinical signs of CVL and that dogs with mild/moderate clinical signs of CVL predominated the sample may be related to the endemic situation of the disease in the city of Barra Mansa and to the fact that these dogs were delivered to the city administration by their owners upon spontaneous demand as dogs which were ill. In the parasitological tests, 79.2% (43/54) of the dogs tested positive in at least one of the techniques, a result that is within the expected range for the average positivity in the literature as well as for the city of Barra Mansa [17,28,29].

We observed a significant difference between the proportion of positivity in dogs with mild/moderate and severe clinical conditions using the K26-LAMP and 18S-qPCR techniques with the SW-kit. The same was observed with the K-26-LAMP technique with SW-DB. In all techniques, the proportion of positives was higher in the group with severe clinical disease, with statistically significant differences being seen in the K26-LAMP and 18S-qPCR techniques with the SW-kit and 18S-qPCR with SW-DB. The median Cts was significantly lower in the severe cases, indicating that severe cases may have a higher parasite load and that this may have influenced the sensitivity of the tests. The target used in cPCR (kDNA) was more sensitive than the others, as it showed a higher number of copies. Perhaps this explains why it was not influenced by the clinical conditions of the studied animals [30]. The purity of the samples extracted by qPCR influenced the results of this technique. The 260/280 ratio values were different between both extraction techniques (*p* < 0.001), indicating contamination with other molecules, such as proteins, phenol, or salts from the SW-DB extraction stage (*p* < 0.001) [31]. The 18S-qPCR tests on these samples were only possible after dilution (1:10) due to the presence of these contaminants and inhibitors.

The conjunctiva is important for the diagnosis of CVL because it is a peripheral lymphoid organ which is affected by *Leishmania* sp. through the dissemination of macrophages [32]. Collecting a sample from the conjunctiva is easy to perform, minimally invasive, and causes less discomfort and less risk to the dog when compared to the collection of other types of samples [33]. Due to the simplicity of the technique, it can reduce time and costs, especially during mass investigations [30]. A recent systematic review showed that conjunctival swab samples are generally 12% better at detecting CVL positivity by PCR than conventional samples, such as bone marrow or lymph nodes, suggesting that ocular structures are reliable sources for diagnosis [11]. In our study, except for one dog in the LAMP technique, all the other dogs with ocular lesions were positive in all diagnostic techniques, indicating that more studies are needed to elucidate the relationship between the presence of ocular lesions and the sensitivity of the techniques.

A previous study testing the DB technique for swabbing the skin lesions of patients suspected of having cutaneous leishmaniasis showed lower sensitivity results with different molecular techniques when compared to samples extracted through a diagnostic kit [16]. The result of this study shows the opposite, a greater positivity in the K26-LAMP and kDNA-cPCR tests in samples extracted through DB, with a significant statistical difference being observed when comparing dogs with mild/moderate and severe clinical conditions. These results may be explained by the different protocols used and the different types of leishmaniasis studied. Another explanation is the parasite load present in the dogs’ conjunctiva, which tends to be higher than the parasite load present in skin lesions in humans, probably due to the decrease in the parasite load resulting from the high intensity of the inflammatory reaction in the skin lesion [16].

The K26-LAMP and kDNA-cPCR techniques showed higher sensitivities in the SW-DB method. In turn, the sensitivity of 18S-qPCR was higher with the SW-kit. The differences between the sensitivities of the SW-kit and SW-DB in the K26-LAMP and 18S-qPCR techniques were statistically significant. Although the number of copies of kDNA minicircles is higher than that of the K26 gene, the LAMP technique is more efficient in amplifying genetic material than PCR. The LAMP technique increases the amount of amplified DNA by up to a billion copies over less than an hour compared to the million copies yielded by PCR [34].

DB executed with kDNA-cPCR and K26-LAMP showed high performance, with sensitivity values of 95.2% and 97.6%, respectively, which are values equal to or higher than other PCR assays for swabs with DNA extracted with a diagnostic kit or phenol–chloroform, whether with kDNA-145bp or other molecular targets [30,35,36]. The tolerance of the LAMP technique to interferents and inhibitors has already been observed in the literature [15]. The cPCR technique, despite being widely used, has limitations regarding standardization and the absence of diagnostic kits, as well as the need for a complex laboratory structure, making LAMP a promising technology that combines practicality, efficacy, and speed in its execution [37,38].

Studies carried out with different samples, molecular targets, primer designs, reagents, DNA extraction methods, and the reading of results for LAMP-CVL show a sensitivity of between 54.2 and 86.2% [35,39,40,41], while, in our study, we obtained a sensitivity of 71.2–97.6%. The standardization of K26-LAMP [23] for use in the diagnosis of human VL presented an analytical sensitivity of 1 fg DNA, a more sensitive result than those obtained in this study, of 100 fg. The difference in sensitivity is related to the different forms of standardization used in the two studies. Although the primers used are the same, the LAMP assay proposed [23] used a longer reaction time (60 min) and a different method of detecting the amplified material (analysis by turbidity caused by the accumulation of magnesium pyrophosphate) than the one used in our study (40 min of reaction and phenol red as an indicator of pH change). The high variation in methodology for LAMP-CVL may be a limiting factor of the technique, reinforcing the need for its standardization in terms of its effective use, considering the various species of *Leishmania* and their genetic variability [23]. 

According to the instructions of the WarmStart^®^ colorimetric kit, negative samples remain pink and positive samples turn yellow due to the color change caused by the acidification of the product, initially having a pH of around 8 that eventually reaches approximately 6. However, in our study, some samples showed an intermediate color (orange/yellow variations), a fact observed by Coelho et al. [42] when evaluating LAMP with a WarmStart^®^ mix for COVID-19. The authors reported that the viral load of the intermediate-colored samples was precisely at the threshold of positive Ct, relating color to viral load. Due to the color variations found, it is suggested that the LAMP tests should be carried out in duplicate or associated with another confirmatory test and/or subsequent development in agarose gel. In this study, the results of the visual analysis were confirmed using agarose gels in all LAMP tests.

The LAMP technique is a rapid and easy-to-use nucleic acid test, but it still has some limitations, such as its dependence on electricity, trained personnel, and equipment [43]. In addition, visual reading should ideally be replaced by the analysis of results using optical devices, reducing the subjectivity of result interpretation. The lack of controls to verify the specificity of the techniques evaluated was a limitation of our study too. However, the observed sensitivity demonstrates great potential in terms of LAMP, considering the ease of sample collection and execution of the technique.

The combination of diagnostic methods to differentiate between sick and non-diseased dogs for CVL has been much discussed and is proving to be a method of effective diagnosis [8]. The combination of less invasive and painless collection, simplified extraction, and simplified amplification can enable a faster, lower-cost, more accessible, and effective form of diagnostic test.

## 5. Conclusions

The results of this study demonstrate the effectiveness of a simple method of DNA extraction through boiling which can be combined with different diagnostic techniques, mainly kDNA-cPCR and K26-LAMP, in clinical samples for less-invasive collection and higher levels of comfort for the animal. For dogs with severe clinical signs of CVL, the combination of DB with K26-LAMP is indicated. The results of this study are unprecedented in the scientific literature and could be used as a starting point for new, more robust, multicenter trials aimed at validating diagnostic methods to be used in public health.

## Figures and Tables

**Table 1:** Clinical score assigned to each item on the clinical form used to record the health observations and clinical examinations of the dogs studied. Dogs seropositive for CVL from Barra Mansa—RJ between 2020 and 2023. Adapted from Chagas et al. [4].

		Score Awarded	
Category	0	1	2
Ectoparasites	Absent	Fleas	Fleas and ticks
Skin	No changes	Skin lesions/ Local alopecia/ Local dermatitis	Ulcerated skin Lesion/generalized Alopecia/generalized Dermatitis
Body condition	Normal	Thin/obese	Cachectic
Onychogryphosis	Absent	—	Present
Ophthalmic changes	Absent	Present (one-sided/light)	Severe (bilateral/purulent)
Lymphadenomegaly	Normal	Local	Regional/generalized
Splenomegaly	Absent	—	Present
Hepatomegaly	Absent	—	Present
Limb edema	Absent	Present	—
Paresis of hindquarters	Absent	Present	—
Mucous membranes	Normal	Pale	Hyperemic
Dehydration	Absent	Mild	Severe
Epistaxis	Absent	Mild	Severe

Group 1: no clinical signs of CVL, with a score of zero. Group 2: mild to moderate clinical signs, between 1 and 7 points. Group 3: severe clinical signs, between 8 and 13 points.

**Table 2:** Clinical signs of dogs seropositive for CVL admitted to the study. Dogs from Barra Mansa, RJ, from 2020 to 2023.

Clinical Observation	n/N	%
Ectoparasites	25/43	58.1
Dermatitis (and/or ulcers)	20/43	46.5
Alopecia	19/43	44.1
Poor body condition	18/43	41.8
Skin scaling	18/43	41.8
Splenomegaly	16/43	37.2
Onychogryphosis	15/43	34.8
Lymphadenopathy	15/43	34.8
Ophthalmic lesions	12/43	27.9
Changes in the mucous membranes	9/43	20.9
Dehydration	6/43	13.9
Paresis of hindquarters	2/43	4.6
Epistaxis	1/43	2.3
Hepatomegaly	1/43	2.3
Limb edema	1/43	2.3

**Table 3:** Median (±max/min) of DNA quantification by nanodrop, featuring a purity ratio of 260/280 and a cycle threshold Ct/PCR according to the extraction method. Clinical samples of dogs seropositive for CVL from Barra Mansa, RJ, Brazil between 2020 and 2023.

Clinical Sample	Quantification (ng/µL)	Ratio 260/280 (nm)	Ct
	(Median ± Max–Min)	(Median ± Max–Min) (~1.80)	(Median ± Max)
SW-DB	299.55 (626.3–25.9) *	0.58 (2.12–0.41) *	29.34 (20.88–33.52) *
SW-kit	22.65 (161.1–4.7) *	1.86 (2.20–1.30) *	27.70 (17.41–32.26) *

ng/µL: nanograms/microliters. nm: nanometer. SW-DB: swab with DNA extracted through boiling method. SW-kit: swab with DNA extracted using a commercial kit. Ct: cycle threshold. * *p*-value < 0.001 (Wilcoxon test).

**Table 4:** Sensitivity of K26-LAMP, kDNA-cPCR, and 18S-qPCR techniques according to the extraction method. Clinical samples of dogs seropositive for CVL from Barra Mansa, RJ, Brazil between 2020 and 2023.

SW-Kit				SW-DB			
	Sensitivity%	(n/N)	95% CI	Sensitivity%	(n/N)	95% CI	*p*-Value
K26-LAMP	72.1	31/43	56.3–84.7	97.6	41/42	87.7–99.6	0.006
kDNA-cPCR	81.4	35/43	66.6–91.6	95.2	40/42	84.2–98.7	0.109
18S-qPCR	80.5	33/43	61.3–88.2	57.1	24/42	40.9–72.2	0.006

n: number of positive samples. N: total of analyzed samples. CI: confidence interval. SW-DB: swab with DNA extracted through boiling method. SW-kit: swab with DNA extracted using a commercial kit. K26-LAMP: loop-mediated amplification with target K26. kDNA-cPCR: conventional PCR with a kDNA target. 18S-qPCR: real-time PCR with a 18S rDNA target.

**Table 5:** Sensitivity rates of K26-LAMP, kDNA-cPCR, and 18S-qPCR techniques considering extraction methods according to clinical signs. Clinical samples of dogs seropositive for CVL from Barra Mansa, RJ, Brazil between 2020 and 2023.

SW-Kit							
Mild/Moderate				Severe			
	Sensitivity %	(n/N)	95% CI	Sensitivity %	(n/N)	95% CI	*p*-Value
K26-LAMP	57.7	15/26	36.9–76.7	93.8	15/16	69.7–99.8	0.015
kDNA-cPCR	73.1	19/26	52.2–88.4	93.8	15/16	69.7–99.8	0.127
18S-qPCR *	68.0	17/25	46.5–85.1	100	15/15	78.1-100	0.016
**SW-DB**							
Mild/Moderate **				**Severe**			
	**Sensitivity %**	**(n/N)**	**95% CI**	**Sensitivity %**	**(n/N)**	**95% CI**	***p*-Value**
K26-LAMP	96.0	24/25	79.6–99.9	100	16/16	79.3–100	0.512
kDNA-cPCR	96.0	24/25	79.6–99.9	93.8	15/16	69.7–99.8	1.000
18S-qPCR	36.0	9/25	18.0–57.5	93.8	15/16	69.7–99.8	<0.001

n: number of positive samples. N: total analyzed samples. CI: confidence interval. SW-kit: swab with DNA extracted through a commercial kit. SW-DB: swab with DNA extracted through the boiling method. K26-LAMP: loop-mediated amplification with K26 target. kDNA-cPCR: conventional PCR with kDNA target. 18S-qPCR: real-time PCR with 18S rDNA target. * Two cases of qPCR not performed (one in the mild/moderate group and one in the severe group). ** One case in the mild/moderate group did not have LAMP, cPCR, or qPCR performed. Only one dog presented 0 in the clinical score and was not included in the statistical analysis.

## Data Availability

The raw data supporting the conclusions of this article will be made available by the authors on request.
